# On Inflammation and Suppuration of the Spleen

**Published:** 1830-01-01

**Authors:** 


### On Inflammation and Suppuration of the Spleen. By A. Raikem, M.D.

Enlargements of the spleen, tubercles on its peritoneal coat and capsule or even in its interior, cartilaginous-like induration of its surface, and softening of its texture, are alterations of frequent occurrence when compared with the formation of abscess in the organ. Those viscera that contain most cellular membrane are always most prone to the formation of abscess, which is thus more common in the lungs than in any other parenchymatous structure. The spleen being chiefly composed of blood-vessels does not evince the same disposition to the formation of pus as the pulmonary tissue. On the contrary, suppuration in its substance is so rare as to make any well attested histories of the affection worth the perusal of the scientific physician. The following interesting case is published in the Repertoire d'Anatomie, tome VII. 1829, by Dr. Raikem, of Volterra.

*Case.* Louis Gazzani, ætatis 20, a book-keeper, of sallow complexion but strong constitution, was addicted to indulgence in spirits, especially rum and punch, and had been affected with syphilitic symptoms. After complaining for some weeks of pain in the left hypochondrium, he was attacked with gastric symptoms in the early part of June, 1829, for which he took a mineral water that vomited and purged him briskly. On the following day Dr. Raikem was summoned to the patient, whom he found suffering from deep pain in the left hypochondrium increased upon pressure—pulse frequent, hard, and jerking—skin hot—tongue covered with a yellowish fur—nausea—loss of appetite—alvine evacuations yellow and liquid. In the evening there was a paroxysm preceded by cold in the feet, and on the following morning there was a remission of the symptoms, though the fever continued and the pulse was still strong and sharp. On the following days the fever appeared to assume a remittent type, and after the employment of cooling drinks and emollient glysters our author prescribed the quinine. The fever still continued, the pulse was upwards of 90, hard and jerking, there was nausea and frequent vomiting, the stools were liquid, and the strength declined. Besides these symptoms the patient complained of lancinating pains increased on pressure in the left hypochondrium, and a hard tumour could be felt in that region. The complexion now became more yellow and the symptoms experienced an exacerbation every evening, all which circumstances led our author to pronounce that the case was one of inflammation of the spleen terminating in suppuration.

Warm baths, fomentations, lavements, a blister to the left side, mercurial inunction, the internal use of calomel and digitalis, and refrigerant drinks with acetate of potass were the means pursued. The tumour, notwithstanding, made progress, extended to the linea alba and umbilicus, was the seat of violent pain, and became softer in proportion with its growth. Having reached this extent, it again diminished in volume, and the pain was considerably mitigated, but the patient began to suffer from a sense of intolerable dragging and constant lancinating pains in the left lumbar region, where œdematous swelling and deep fluctuation were discovered. This was the spot where the blister had been applied, and the slightest pressure exasperated the sufferings. Thinking that the pus had escaped from the spleen and passed behind the peritoneum and between it and the muscles, to this situation, Dr. Raikem requested M. Nic. Bianchi, an able surgeon, to meet him in consultation, and consider the propriety of evacuating the matter by an incision. For reasons which we need not detail, it was not deemed advisable by the consultants to run the risk of the operation, to which the patient likewise refused to submit. The pains in the lumbar region soon ceased to be severe, but the fever and other symptoms grew rapidly worse; the belly was blown up, and painful on the left side from the hypochondrium to the groin; a hard, very painful, and circumscribed tumour appeared in the latter situation; the breathing became hurried, laborious, and attended with pain on inspiration; there was frequent dry cough; difficulty of lying on either side; pulse rapid and weak; partial sweats on the surface of the body; prostration of strength; diarrhœa; and, on the 25th of July, about two months after our author's first visit, the unfortunate patient expired.

*Sectio Cadaveris.*—In the left side of the chest, were upwards of two pints of yellow serum with flakes of lymph. The pleura was coated with a layer of false membrane, and various bands of recent lymph connected the pleura costalis and pulmonalis with each other. The lobes of the lung were adherent, but the pulmonary texture itself was sound. The right side of the chest shewed nothing unusual; there were some ounces of yellow serum in the pericardium.

On opening the abdomen, some "elastic fluid" escaped. The liver was healthy; the stomach and intestines appeared to be so too, but they were not laid open. The superior portion of the descending colon, and the great end of the stomach, were closely united to the spleen. This latter viscus was double its ordinary size, and of vermilion colour on its borders. The peritoneum opposite its posterior surface was raised by a whitish, thick, purulent, and very fetid fluid, which was found to have issued from the cavity of a vast abscess in the substance of the spleen. From this it had extended between the peritoneum and the abdominal muscles as far as the kidney, the spine, and even as low as the crural ring. The extensive fetor of the matter prevented any more minute examination.

Dr. Raikem makes some valuable remarks on the preceding case, which he illustrates by others, collected with great labour and research from the older and most approved writers. In order to render the present article more complete, we shall take the liberty of borrowing a few of our author's facts. Though *Haller* and *Baillie* agree in pronouncing suppuration, i. e. abscess, of the spleen to be rare, still several instances are recorded in the Répertoire de Médicine pratique of *Plouquet*, and our author has sometimes observed it in the bodies of patients who have suffered from intermittent fever. *Frank* enumerates accurately the different modes by which an abscess in the spleen may be evacuated;—viz. by opening into the stomach, when it produces vomiting of bloody pus; into the colon, with diarrhœa and discharge of the same kind of matter by stool; into the left side of the chest, or even into the lung, with the symptoms of phthisis pulmonalis; into the abdominal cavity, or finally into the cellular texture between the peritoneum and dorsal, or abdominal muscles. In the last mentioned case, the patient most commonly dies with the ordinary symptoms of destructive suppuration, but nevertheless there are instances of recovery on record. *Fantoni* mentions that an abscess of the spleen discharged itself at the umbilicus, but the patient recovered, was brought to bed of a male infant, and did not die until five years afterwards. On opening the body, no vestige of the spleen remained, and the neighbouring parts were united together by cicatrices, in the usual situation of the missing organ. In another instance, related by *Grottanelli;*—a Franciscan Monk, ætatis 67, who had suffered from intermittent fever, experienced pain in the region of the spleen which was hard and painful. In about three weeks a tumour appeared with fluctuation, and threatening of suppuration, and three days afterwards the abscess burst, and discharged matter of "a steatomatous and albuminous character." During the first three days, about four pints of the fluid were discharged in the twenty-four hours, a probe introduced obliquely upwards to the height of four inches occasioned no pain, but when carried downwards pain was complained of. The patient recovered with permanent enlargement of the spleen, and lived for two years, when he died of fever.

With regard to the effusion of the contents of a splenic abscess into the peritoneal cavity, we need scarcely cite cases to prove its fatal consequences. The following, however, is so well marked an example that we cannot forbear giving the brief particulars.

A young female orphan about ten years old, who had suffered from obstinate intermittent fevers, was admitted into the hospital of Pitigliano on the 16th of October, 1820. She was much emaciated, but presented an unusual tumefaction of the abdomen, which at first was taken for ascites, but proved in a short time to depend on an enlargement of the spleen which extended from the left to the right hypochondrium, and passed lower than the umbilicus. The patient laboured under fever, with occasional dry and very troublesome cough, irregularity of the bowels, and frequently retention of urine. In spite of the remedial measures employed the fever and the pain increased in violence till the 27th of October, when suddenly the sufferings ceased, the tumour disappeared almost entirely, and the patient could lie on either side. Next day she insisted on getting up notwithstanding every remonstrance, and that night the belly swelled greatly, dyspnœa supervened, and she sank on the morning of the 30th. On examination after death the peritoneum was found injected, and the abdominal cavity contained upwards of two pints of limpid serum; gelatinous-like, concrete matter was found in the pelvis; and the same invested the surface of the spleen. The colon, the great end of the stomach and the contiguous peritoneal surface of the spleen, which was about nine inches in length and five in breadth. The anterior and inferior part was in a state of suppuration and exhibited a large rent with destruction of substance extending to its posterior portion. The sound part of the viscus was covered with false membrane, and the part of the diaphragm in contact with the spleen was inflamed. Several of the mesenteric glands were obstructed; the omentum was nearly destroyed. The left lung was inflamed in its inferior part and studded with a number of softened tubercles. *Grottanelli, de Splenite.*

But although so generally fatal, cases have been related by authors with the view of shewing that rupture of the spleen or of an abscess into the belly is not so invariably. *Grottanelli* in the work already cited adduces the following.

A man residing in the environs of Aquapendente, about twenty years of age, who had suffered from venereal affections, went in 1810 to reap in the commune of Sienna and was seized with a double tertian fever. The spleen also swelled and grew very painful, but in proportion as the fever subsided under proper treatment the spleen returned to its natural size. Some time afterwards, having travelled on foot in rainy weather, he was attacked with intermittent catarrhal fever, about the fourth day of which the spleen again swelled and grew painful. Nausea, vomiting, pain in the left shoulder, and slight cough succeeded—the pain in the splenic region was relieved by fomentations, whilst the tumefaction remained—the fever was quotidian and hectic in its character, and the patient now refused to submit to any further treatment, and had recourse to wine. About a month after this he received a violent kick in the belly, which brought him to the ground and he was carried home insensible. He recovered his faculties in the course of a quarter of an hour, and declared that at the instant of receiving the blow, he felt a most horrible pain in the region of the spleen. He could only lie upon the back with the chest much raised, and on looking for the tumour in the left hypochondrium it *could no longer be discovered.* In the course of twenty hours from the infliction of the injury the skin was universally covered with spots like the ecchymoses of scurvy, and the urine became so loaded that it resembled the alvine dejections of an infant. This state of the urine continued for three months when all the pain and fever had entirely vanished, and seven years after this occurrence *Grottanelli* found the man at Civita-Vecchia enjoying excellent health. He adds that he pointed out the individual to his companions, *quasi tabulam à naufragio ereptam,* like a plank saved from a shipwreck.

The next case is likewise to be found in Grottanelli's work, in which it is inserted by Professor Bazelotti. Antoine Columbi, of delicate constitution, laboured under swelling and occasional pain in the spleen, and appeared to be disposed tò phthisis. One day, whilst in the ènvirons of Monterotondo, he received a violent kick from an unruly horse in the left iliac and hypochondriac regions, which felled him to the ground and deprived him of his senses. Being carried home he complained of very violent pain in the region of the spleen, fever supervened, and the surgeon who attended bled and purged him freely. For several days the urine was bloody and thick, but the patient in the end recovered perfectly.

Whether this is to be considered as another *plank* saved by chance or by skill, is more than the doctor ventures to inform us. We cannot consider it a satisfactory instance of recovery after effusion of the contents of the spleen into the abdominal cavity, neither do we think the first case conclusive, although more difficult to get over than the present. It is always dangerous to admit such important pathological points as that now involved, unless their correctness be fully established by the scalpel after death. At the same time the cases are not devoid of interest, and we think that the paper is worth the attention of our readers, since it comprises more than is commonly known on the subject of inflammation and suppuration of the spleen.

---

